# A Berberine-Loaded *Bletilla striata* Polysaccharide Hydrogel as a New Medical Dressing for Diabetic Wound Healing

**DOI:** 10.3390/ijms242216286

**Published:** 2023-11-14

**Authors:** Zhengbo Hu, Kai Zhao, Xingcan Chen, Mingyuan Zhou, Yuchi Chen, Xiaoqing Ye, Fangmei Zhou, Zhishan Ding, Bingqi Zhu

**Affiliations:** School of Medical Technology and Information Engineering, Zhejiang Chinese Medical University, Hangzhou 310053, China

**Keywords:** diabetic wound, *Bletilla striata* polysaccharide, berberine, hydrogel

## Abstract

The healing process of a diabetic wound (DW) is often impeded by a series of interrelated factors, including severe infection, persistent inflammation, and excessive oxidative stress. Therefore, it is particularly crucial to develop a medical dressing that can address these issues simultaneously. To this end, different ratios of *Bletilla striata* polysaccharide (BSP) and berberine (BER) were physically blended with Carbomer 940 (CBM940) to develop a composite hydrogel as a medical dressing. The BSP/BER hydrogel was characterized using SEM, FTIR, rheological testing and other techniques. The anti-inflammatory, antioxidant, and antibacterial properties of the hydrogel were evaluated using cell and bacterial models in vitro. A DW model of ICR mice was established to evaluate the effect of the hydrogel on DW healing in vivo. The hydrogel exhibited excellent biocompatibility and remarkable antibacterial, anti-inflammatory, and antioxidant properties. In addition, animal experiments showed that the BSP/BER hydrogel significantly accelerated wound healing in DW mice. Among the different formulations, the LBSP/BER hydrogel (2% BSP, m_BER_:m_BSP_ = 1:40) demonstrated the most remarkable efficacy. In conclusion, the BSP/BER hydrogel developed exhibited immense properties and great potential as a medical dressing for the repair of DW, addressing a crucial need in clinical practice.

## 1. Introduction

As a chronic metabolic disease, diabetes mellitus exists widely in the world, with patients numbering more than 463 million [1]. The diabetic wound (DW) is a prevalent, costly, and serious complication of diabetes [2,3,4], and it often leads to lower limb amputation, with post-amputation mortality rates ranging from 54% to 79% [5,6]. This not only significantly impacts the lives of patients but also presents a major challenge to the global healthcare system.

Promoting the healing of DWs is a complex task due to a series of interrelated factors. Firstly, the high blood glucose level at the wound site provides a favorable condition for bacterial growth and increases the risk of infection, leading to excessive oxidative stress and inflammation [7]. Secondly, the sustained release of inflammatory factors at the wound lesion site and the impaired transition of M1 macrophages to M2 macrophages contribute to the development of chronic inflammation [8]. Concurrently, the presence of neutrophils and macrophages persists, resulting in the continuous production of reactive oxygen species (ROS) and the subsequent excessive oxidative stress damage [9]. Therefore, developing targeted treatments addressing these issues is a promising strategy for managing DWs.

In order to develop comprehensive and multifunctional dressings for DW treatment, numerous dressing preparation strategies have been proposed. Due to their antibacterial properties, metal nanoparticles have found applications in the development of dressings, such as silver nanoparticle hydrogel [10,11] and Cu nanoparticle-based hydrogel [12]. These dressings have demonstrated effectiveness in the treatment of diabetic wounds (DWs). However, it is essential to consider the cytotoxicity associated with metal nanoparticles. On the other hand, safe and non-toxic natural materials and their derivatives present promising candidates. Examples include natural cordycepin/chitosan hydrogel [13], methacrylic anhydride (MA)-modified gelatin hydrogel [14], and gallic acid-grafted quaternized chitosan (GA-QCS)/oxidized hyaluronic acid (OHA) hydrogel [15], etc. These dressings improve the biocompatibility and fulfill a variety requirement for wound dressings; however, their preparation methods tend to be complex and costly. Therefore, the development of more comprehensive and practical DW dressings remains a challenge.

*Bletilla striata* polysaccharide (BSP) is the primary component of *Bletilla striata* and serves as a highly biocompatible natural glucomannan [16]. It has been proven to greatly facilitate epithelial regeneration, neovascularization, and collagen deposition during the repair process [17]. These properties make BSP a promising candidate for the preparation of DW dressings. Our previous research and development of a BSP composite sponge dressing has substantiated its efficacy in promoting wound healing [18,19]. Berberine (BER) is the main component of *Coptis chinensis*, which has good safety in clinical use [20]. It exhibits a broad spectrum of biological activities, including antibacterial, anti-inflammatory, antioxidant, and antidiabetic properties [20,21].Therefore, the synergy between BSP and BER can effectively address DWs from various angles, providing comprehensive management and treatment.

The hydrogel dressing, with its three-dimensional network structure, closely resembles the natural extracellular matrix in both physical and functional aspects. This makes it an excellent choice for managing DWs [22,23,24]. One of its key advantages is its high loading capacity, which allows for the incorporation of various active substances, resulting in more comprehensive pharmacological activity [24,25]. Carbomer (CBM)-based hydrogel demonstrates great promise as a hydrogel medical dressing due to its affordability, straightforward preparation process, remarkable biocompatibility, hydrophilicity, and adhesion properties [26]. Therefore, leveraging CBM-based hydrogel to encapsulate BSP and BER holds great potential for the treatment of DWs.

**Scheme 1:**
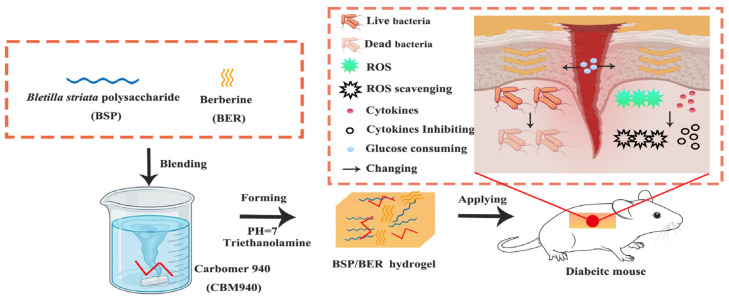
Schematic illustration of the simple preparation method for *Bletilla striata* polysaccharide (BSP)/berberine (BER) hydrogel and its mechanism for promoting diabetic wound healing.

In this study, given the multifaceted nature of the factors influencing DWs, a CBM940-based natural hydrogel loaded with BSP and BER was meticulously designed and prepared. The properties of the hydrogel were thoroughly tested, yielding promising results. The findings indicated that the hydrogel effectively creates an ideal moist healing environment, mitigates bacterial infection, shows anti-inflammatory and antioxidant properties, and stimulates vascular and epithelial regeneration. In a short, this hydrogel holds considerable potential in promoting the healing process of DWs (Scheme 1).

## 2. Results and Discussion

### 2.1. Characterizations of BSP/BER Hydrogel

**Figure 1:**
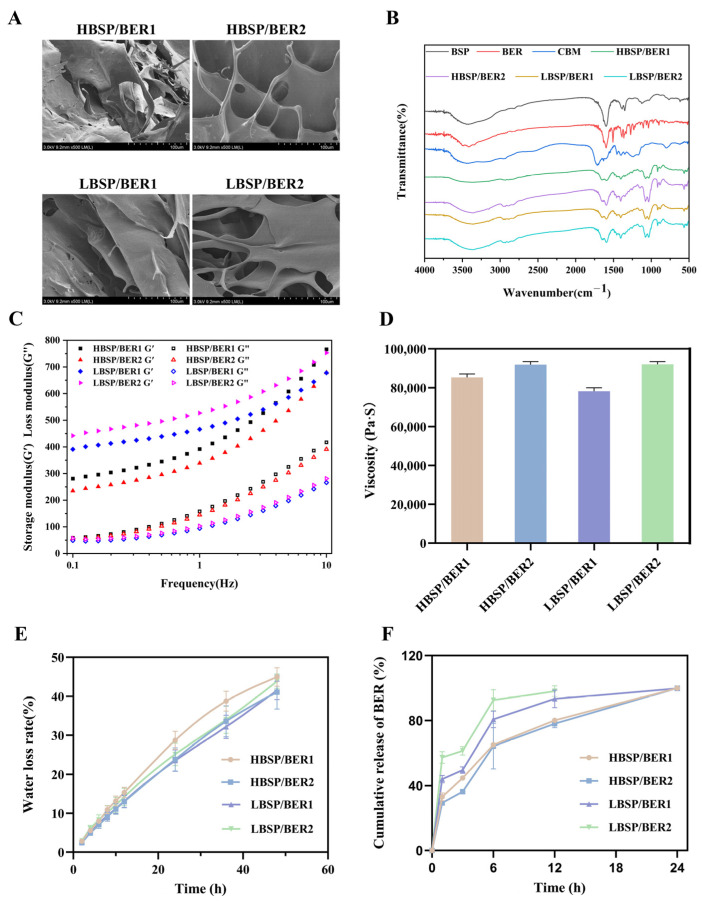
Characterization of BSP/BER hydrogel. (**A**) Representative SEM images of the hydrogel. (**B**) FTIR spectra of BSP, BER, CBM and BSP/BER hydrogel. (**C**) The storage (G′) and loss (G″) moduli of the BSP/BER hydrogel were measured over a frequency range of 0.1–10 Hz. (**D**) Viscosity of the BSP/BER hydrogel. (**E**) The water loss rate of BSP/BER hydrogel within 48 h at 37 °C. (**F**) Cumulative release of BER from the BSP/BER hydrogel in PBS (pH = 7.4) within 24 h at 37 °C.

Based on the material ratios presented in Table 1, four different hydrogels were prepared and subsequently characterized. As demonstrated in Figure 1A, the hydrogels obtained exhibited a multi-layered loose structure, which was beneficial to the gas exchange on the wound surface [27].

The FTIR spectra are shown in Figure 1B. In the BSP spectrum, the absorption of the stretching vibration of the -OH band was at 3420 cm^−1^. The peak at 2819 cm^−1^ was attributed to -CH. The tensile vibration of -COOH was observed at 1732 cm^−1^ [27]. The absorption band at 1126 cm^−1^ was the asymmetric tensile vibration of -C-O-C- in pyran [28]. The 872 cm^−1^ and 764 cm^−1^ absorption bands were derived from β-glucose and mannose residues, respectively. Regarding the BER, in addition to the tensile vibration of -OH at 3420 cm^−1^ and the vibration of -CH at 2845 cm^−1^, the absorption bands at 1597 cm^−1^ and 1507 cm^−1^ corresponded to the vibration of the benzene ring skeleton [29]. The absorption bands of CBM940 at 3446 cm^−1^, 2959 cm^−1^, and 1716 cm^−1^ corresponded to the vibrations of -OH, -CH, and -COOH, respectively [30]. The spectra of the BSP/BER hydrogel exhibited characteristic peaks of BSP, BER and CBM940. However, the absorption representing -COOH near 1700 cm^−1^ appeared weaker in the hydrogel, which indicated that the formation of the hydrogel was mostly due to the stable polymer structure formed by the neutralization of the carboxyl groups in the CBM940 (Table 2).

To investigate the rheological properties of the hydrogels, the storage modulus (G′) and loss modulus (G″) of the BSP/BER hydrogels were measured in the frequency range of 0.1–10 Hz. The results, shown in Figure 1C, indicated that the G′ values of all the hydrogels were higher than those of the G″, demonstrating the viscoelastic solid behavior and stability of the BSP/BER hydrogels [31,32]. Moreover, the G′ increased with the frequency, indicating a highly dynamic network within the hydrogels [33].

To effectively repair DWs, hydrogel must possess specific key characteristics, such as proper viscosity, good water retention, and stable drug release ability. The BSP/BER hydrogels used in this study demonstrated a range of viscosity values, with the highest viscosity of LBSP/BER2 at 92,069 mPa·s^−1^ and the lowest of LBSP/BER1 at 78,208 mPa·s^−1^ (Figure 1D). This viscosity enables the hydrogel to effectively adhere to the wound and tissue, providing a long-term healing environment [34]. In the water retention experiment, it was observed that as the time progressed, the hydrogels experienced a gradually increased water loss rate (Figure 1E). During the 48 h experiment, there was no significant difference in the water loss rate observed between these hydrogels. After 48 h, it was able to maintain over 60% moisture content, indicating that it has good water retention capacity, effectively preventing rapid water loss [32]. Compared to other CBM-based hydrogels of the same type, the BSP/BER hydrogel exhibits a significantly lower water loss rate of only 10% within a 15 h period, which is notably less than the 30% observed in other hydrogels [30]. Figure 1F displays the drug release curve of the BSP/BER hydrogel. In PBS at 37 °C, the cumulative drug release increased over time, with the highest increasing rate occurring within the first 6 h, after which the rate gradually slowed down. This indicates that the majority of the BER in the hydrogel was released into the PBS within 6 h. However, as the concentration of BER in the solution increased, the release rate slowed down. Among the different hydrogels, the drug release rate of the HBSP/BER hydrogel was slower compared to the LBSP/BER hydrogel. This difference in release rate could be attributed to the varying cross-linking strength resulting from different BSP contents.

### 2.2. Biocompatibility of BSP/BER Hydrogel

**Figure 2:**
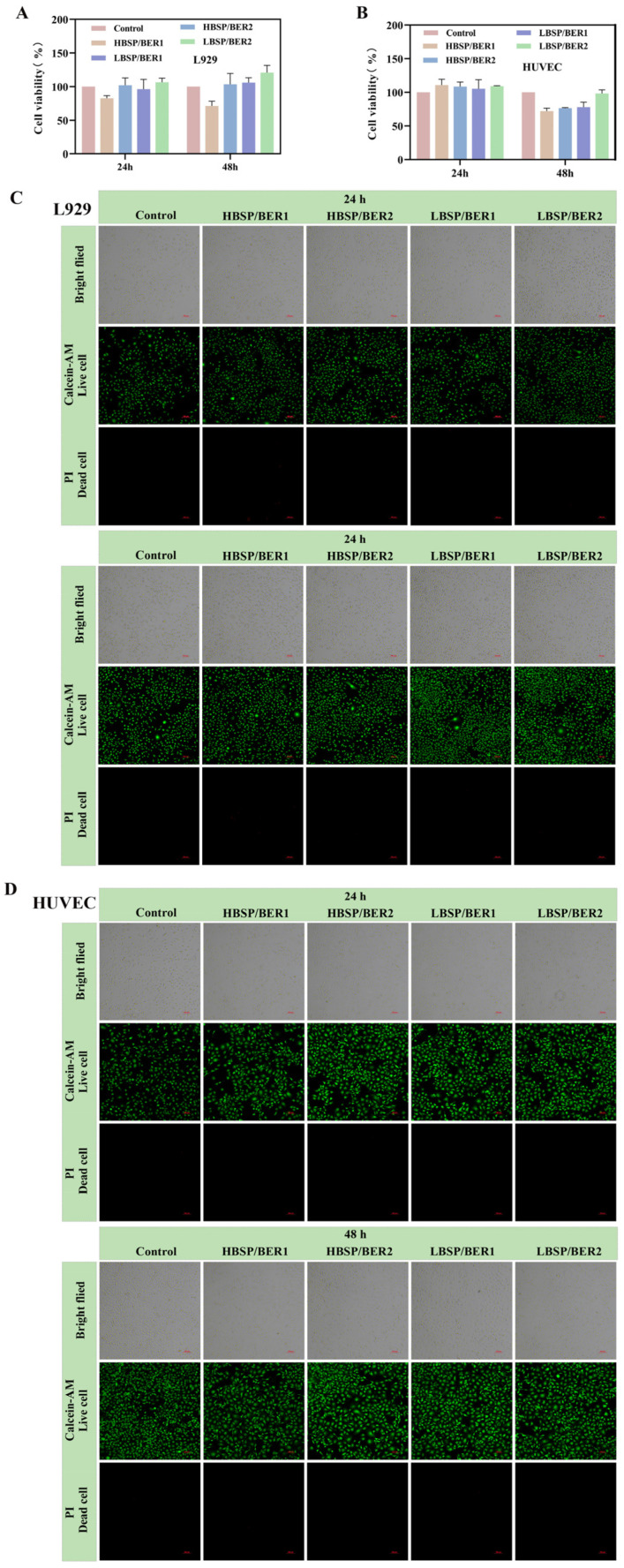
Cytocompatibility of the hydrogel: cell viability of L929 cells (**A**) and HUVEC cells (**B**) treated with BSP/BER hydrogel extracts after 24 h or 48 h; live–dead cell staining of L929 cells (**C**) and HUVEC cells (**D**) treated with BSP/BER hydrogel extracts after 24 h or 48 h. Green: live, red: dead, scar bar: 100 μm.

Superior biocompatibility serves as the core requirement for hydrogel dressings intended for the purpose of healing [35]. In order to confirm the biocompatibility of BSP/BER hydrogel, mouse fibroblasts (L929 cells) and human umbilical vein endothelial cells (HUVEC cells) were selected for testing. The results of the CCK8 experiments are presented in Figure 2A,B. After incubating L929 cells or HUVEC cells with hydrogel extract, the cell viability rates for all the BSP/BER hydrogel groups were higher than 70% at 24 and 48 h, indicating that the BSP/BER hydrogel was non-cytotoxic. The results of the live/dead staining further confirmed that the BSP/BER hydrogel treatment resulted in mostly living cells (green) and few dead cells (red), with no significant change in cell morphology (Figure 2C,D). These findings demonstrate the good cytocompatibility of the BSP/BER hydrogel.

**Figure 3:**
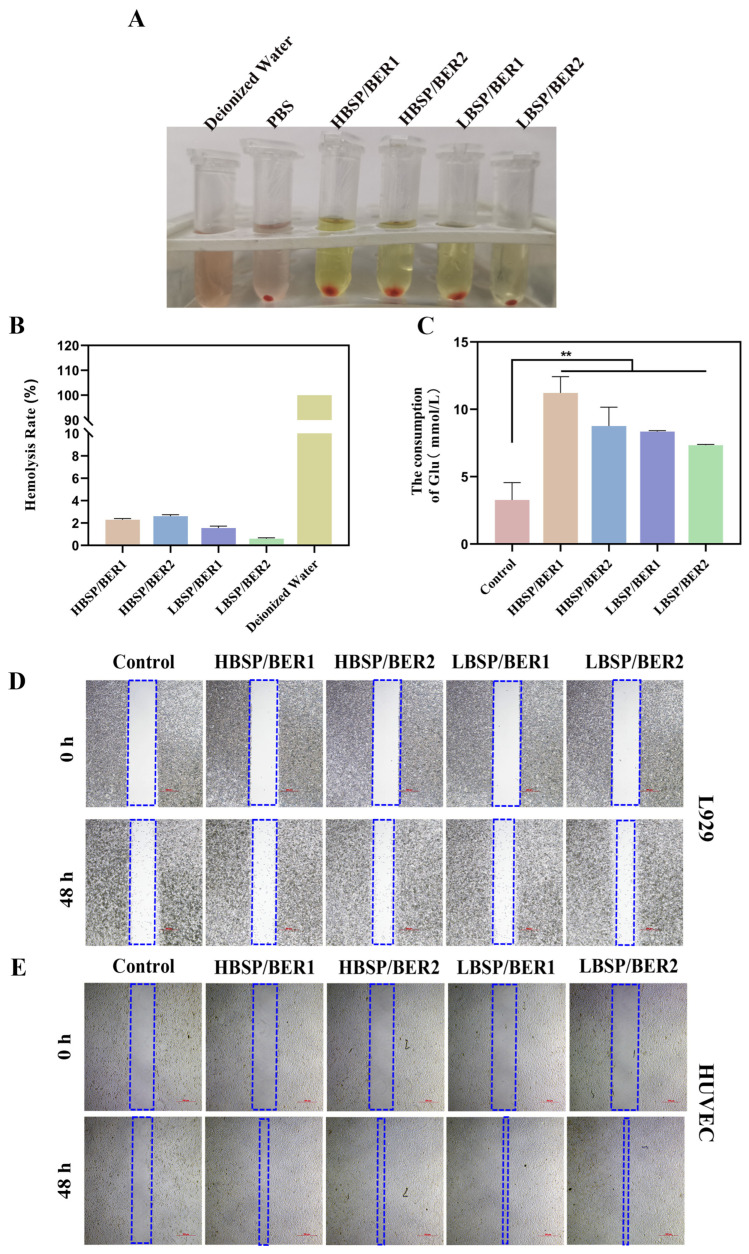
Hemolytic photographs (**A**) and ratio (**B**). (**C**) Effect of hydrogel on glucose consumption of L929 cells. L929 cell (**D**) and HUVEC cell (**E**) migration. The blue frame: schematic diagram of the middle free area of cell, scar bar: 500 μm. Data presented as mean ± SD, *n* = 3. Statistically significant: ** *p* < 0.01.

Additionally, the results of the blood compatibility tests showed that there were aggregated red blood cells at the bottom of the sample after adding PBS solution or BSP/BER hydrogel extracts, and no significant difference in the supernatant color was obtained among the different hydrogels (Figure 3A). After quantitative analysis (Figure 3B), the hemolysis rate of the BSP/BER hydrogel was less than 5%, indicating the prominent hemocompatibility of the BSP/BER hydrogel.

### 2.3. Glucose Consumption and Migration Experiment

The main reasons for the difficulty in healing DWs are the inhibition of the proliferation and migration of fibroblasts, as well as persistent bacterial infection induced by a high glucose environment in the wound area [36]. Therefore, reducing the local glucose levels has gradually become a viable option for promoting the healing of such wounds. In the glucose consumption experiment, L929 cells treated with BSP/BER hydrogel showed significantly higher glucose consumption compared to the control group (Figure 3C). Furthermore, the consumption of glucose exhibited an upward trend with the augmentation of the BER content within the hydrogel. Evidence from cell line and animal studies has confirmed that BER possesses a hypoglycemic effect; therefore, the BER in the hydrogel may be the possible reason for the increment of the glucose consumption [37]. This may be attributed to the activation of glucose transporter GLUTI in L929 cells by BER [38]. These findings demonstrate the potential of BSP/BER hydrogel in reducing local blood glucose levels in the wound region.

The migration experiment conducted on HUVEC and L929 cells is illustrated in Figure 3D,E. The coverage areas of the BSP/BER hydrogel groups were significantly greater compared to the control group, indicating that the prepared hydrogels effectively promoted cell migration. Due to BSP’s distinctive property of promoting cell migration [28,39,40], this effect of the BSP/BER hydrogels could be attributed to the presence of BSP. However, the lack of a significant difference in the ability to promote cell migration among the different groups could be due to the fact that a high level of BSP content was used in the preparation of the hydrogels and limited the BSP content differences presented among different prepared hydrogels.

### 2.4. Antioxidant and Anti-Inflammation Properties of Hydrogel

**Figure 4:**
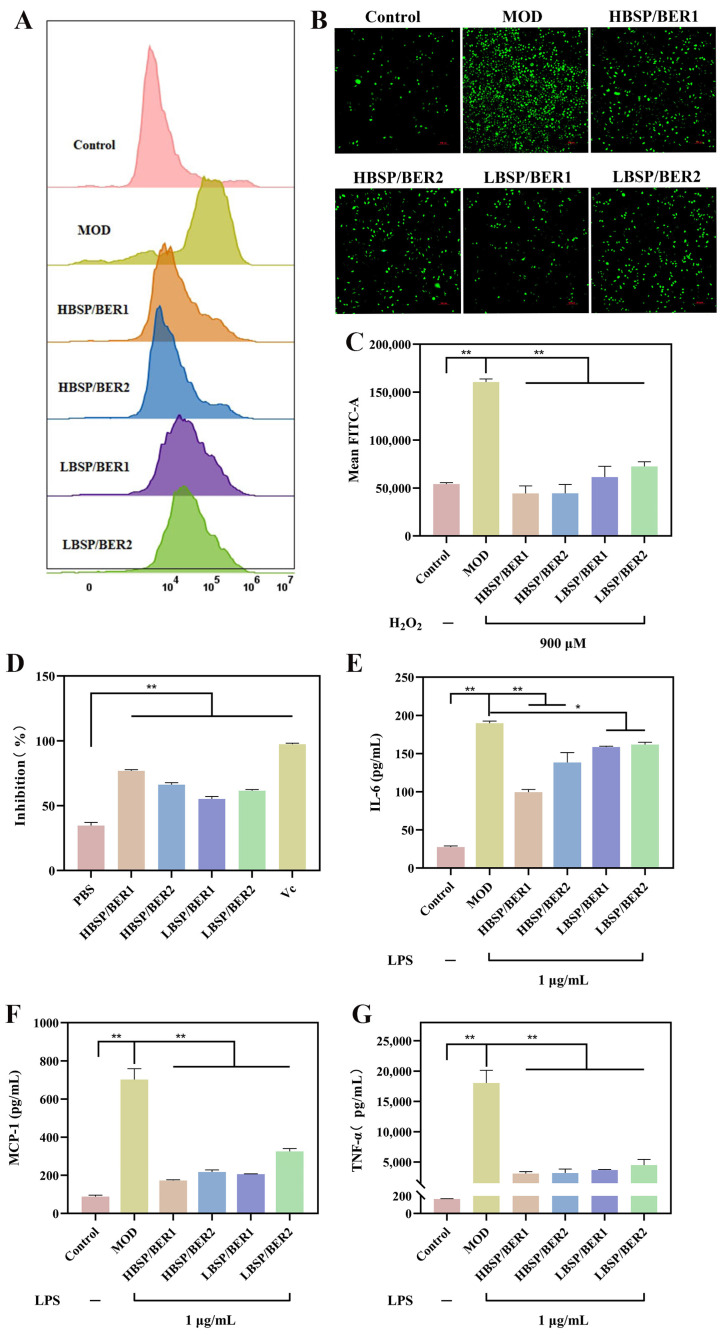
(**A**) Flow cytometry of intracellular reactive oxygen species (ROS) in L929 cells. Fluorescent images. Scar bar: 500 μm (**B**) and mean FITC in L929 cells (**C**). (**D**) Hydrogel scavenging on DPPH. Effects of different hydrogel extracts on interleukin (IL)-6 (**E**), monocyte chemotactic protein (MCP)-1 (**F**), and tumor necrosis factor (TNF)-α (**G**) factors in lipopolysaccharide (LPS)-induced RAW 264.7 cell supernatant. Data presented as mean ± SD, *n* = 3. Statistically significant: * *p* < 0.05, ** *p* < 0.01.

Reactive oxygen species (ROS) play a significant role in the wound healing process. However, in the case of DWs, there is often an excessive amount of oxidative stress in the tissue, leading to the production of a large number of ROS. This, in turn, hinders the wound healing process [41]. To verify the effectiveness of BSP/BER hydrogel in scavenging intracellular ROS, H_2_O_2_ was utilized to stimulate L929 cells and induce oxidative stress, thereby simulating the ROS environment of a DW [42]. The untreated cells served as the control group, while the cells treated only with H_2_O_2_ served as the model group (MOD). Flow cytometry analysis (Figure 4A,C) revealed that the cells treated with H_2_O_2_ exhibited higher fluorescence intensity, which significantly decreased after the hydrogel intervention. This outcome was further confirmed using a fluorescence microscope (Figure 4B).

Additionally, the DPPH scavenging test was used to evaluate the antioxidant properties of the BSP/BER hydrogels in vitro [43]. Figure 4D shows the DDPH scavenging ability of each group of hydrogels. The PBS group did not show an antioxidant effect, while the VC group (the positive control which added vitamin C) had the strongest scavenging ability in terms of DPPH. In addition, the DPPH clearance rates of all the BSP/BER hydrogels were higher than 50%.

Excessive inflammation has consistently been recognized as a significant obstacle to the healing of DWs. The substantial increase in proinflammatory factors within DWs disrupts the delicate balance between proinflammatory and anti-inflammatory factors, which greatly hampers the process of tissue repair. Consequently, the regulation of inflammatory factors is a crucial factor in promoting the healing of DWs [8,44]. In Figure 4E–G, the expression of inflammatory mediators (interleukin (IL)-6, monocyte chemotactic protein (MCP)-1, tumor necrosis factor (TNF)-α) in mouse RAW 264.7 macrophages (RAW264.7 cells) induced by LPS was significantly increased, although after treatment with BSP/BER hydrogel, the levels of these inflammatory cytokines were significantly inhibited (the untreated cells served as the control group, while the cells treated only with LPS served as the model group (MOD)). In contrast to hydrogels with superior ROS scavenging and excessive proinflammatory factor elimination abilities, the preparation process for BSP/BER hydrogels is relatively simple [42,45]. To sum up, the BSP/BER hydrogels showed excellent anti-inflammatory and antioxidant properties.

### 2.5. Antibacterial Properties of BSP/BER Hydrogel

**Figure 5:**
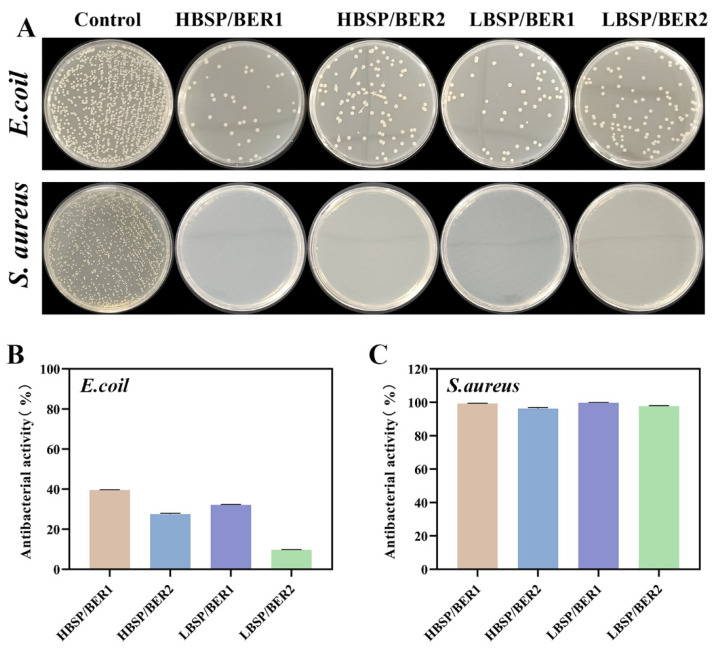
Antibacterial properties of BSP/BER hydrogel. Photographs (**A**) and antibacterial activity of *Escherichia coli* (*E. coli*) (**B**) and *Staphylococcus aureus* (*S. aureus*) (**C**) treated with BSP/BER hydrogel.

Anti-infective treatment is crucial for DW healing; therefore, the antibacterial ability of wound dressings has always been an important research topic [46]. BER, a widely used traditional Chinese medicine ingredient with strong antibacterial properties, is an excellent choice for enhancing the antibacterial ability of hydrogel [29]. The antibacterial properties of BSP/BER hydrogel were evaluated using two types of bacteria (*Staphylococcus aureus* (*S. aureus*) and *Escherichia coli* (*E. coli*). The results are presented in Figure 5A and the antibacterial activity are shown in Figure 5B,C. For *E. coli*, the colony number in the BSP/BER hydrogel groups showed a significant decrease compared to the control group, with a bacteriostatic rate exceeding 20%. And the HBSP/BER1 hydrogel group, which had a higher concentration of BER, exhibited the lowest number of viable colonies and an inhibition rate of approximately 40% against *E. coli*. These results suggest that the bacteriostatic activity of BSP/BER hydrogel against *E. coli* was largely dependent on the berberine content. When it came to *S. aureus*, all the BSP/BER hydrogels demonstrated superior antibacterial properties. No obvious colony was observed on the plate under the influence of any group of BSP/BER hydrogels, and the antibacterial rate was close to 100%. The stronger antibacterial activity of BER against Gram-positive bacteria may explain this phenomenon [47]. In conclusion, the BSP/BER hydrogels demonstrated significant antibacterial effects, especially against *S. aureus.*

### 2.6. In Vivo Diabetic Wound Healing

**Figure 6:**
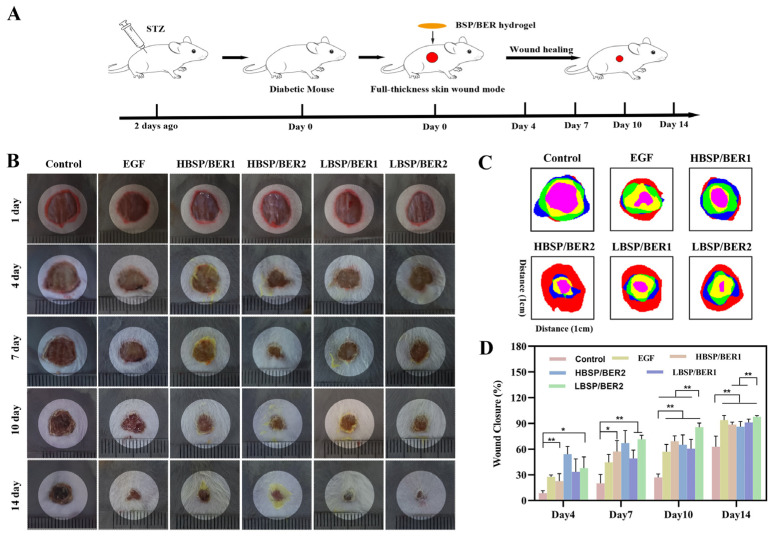
Effect of BSP/BER hydrogel on the healing of diabetic wounds (DWs) in mice. (**A**) Schematic diagram of the establishment of DW mice and treatment with different BSP/BER hydrogels. (**B**) Representative digital photographs of days 1, 4, 7, 10 and 14 under different treatment methods. (**C**) Schematic diagram of DW healing. Red: day 1, blue: day 4, green: day 7, yellow: day 10, pink: day 14. (**D**) Wound closure rate analyzed via Image J. Data presented as mean ± SD, *n* = 5. Statistically significant: * *p* < 0.05, ** *p* < 0.01.

In order to evaluate the ability of BSP/BER hydrogel to promote the healing of DWs, the hydrogel was applied to the full-thickness resected wounds of diabetic mice (Figure 6A–D). After the establishment of the model, the area of mice in each group decreased gradually with the passage of time. However, the control group exhibited a consistently slow wound healing rate compared to the other medication groups at every stage of the actual healing process. In the later stage of wound healing, specifically on the 14th day, the wound healing rate of the mice in the BSP/BER hydrogel group exceeded 90%, which was significantly higher than the approximately 80% rate achieved by the same type of hydrogel [27]. Notably, the group treated with the LBSP/BER2 hydrogel displayed a significantly superior wound healing effect compared to the other groups. In fact, its performance even surpassed that of the positive control group, particularly on the 7th and 14th days, with impressive wound healing rates of 85.8% and 97.7%, respectively. Based on the representative digital photos of the mouse wound, it was observed that the deposition of BER in the LBSP/BER2 hydrogel group was significantly lower compared to the other groups. This excellent degradability may also be one of the reasons contributing to its superior efficacy.

**Figure 7:**
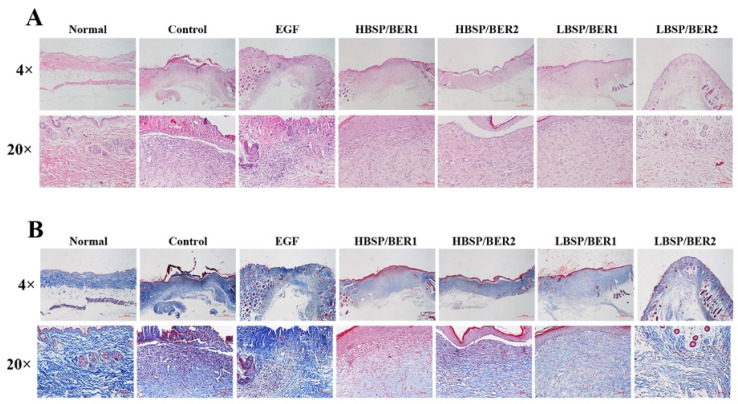
Histology analysis of the wounds treated with EEB/BSP hydrogel at the 14th day. The images of hematoxylin and eosin (H&E) (**A**) and Masson trichrome (**B**) staining under 4× (scar bar: 500 μm) and 20× (scar bar: 100 μm), respectively.

A histological examination was further conducted on the wound tissue after 14 days. The results of the HE staining (Figure 7A) revealed that the control group exhibited significant inflammatory cell infiltration and a thick epidermis after 14 days of treatment. However, the treatment groups showed less inflammatory cell infiltration and a thinner epidermis, with the emergence of new capillaries and hair follicles, in comparison with the control group. These results indicate that the inflammation level of the wound was lower in the treatment groups, with a better degree of healing. The results of the in vivo experiments corresponded to the anti-inflammatory, antibacterial and antioxidant properties of the BSP/BER hydrogel from the in vitro studies, which may have contributed to these positive outcomes [33]. Notably, the LBSP/BER2 group demonstrated the most significant healing among all the hydrogels prepared in comparing with normal skin. Additionally, the results of the Masson trichrome staining (Figure 7B) demonstrated that the collagen distribution was more orderly and denser in the treatment groups, particularly in the LBSP/BER2 group, resembling that of normal skin. These findings confirm the potential of the BSP/BER hydrogel in the healing of DWs and suggest that the LBSP/BER2 hydrogel may have the best therapeutic effect.

In summary, the results of the in vitro experiments indicated that increasing the concentration of BER in the BSP/BER hydrogel within the tested range resulted in enhanced hypoglycemic, anti-inflammatory, antioxidant, and antibacterial effects but also escalated the cytotoxicity. Furthermore, a higher BSP content in the hydrogel demonstrated a faster BER release rate. The in vivo experiment showed that the LBSP/BER2 hydrogel exhibited the most effective promotion of healing. Overall, considering the in vitro and in vivo experimental results, and taking into account the complex and prolonged nature of DW healing, the LBSP/BER2 hydrogel possibly had the greatest potential in promoting DW healing among the four prepared hydrogels. However, further exploration is required to elucidate the specific rules and mechanisms underlying these findings.

## 3. Materials and Methods

### 3.1. Materials

BSP, BER, KBr, and glucose detection kits were purchased from Yuanye Bio-Technology Co., Ltd. (Shanghai, China). CBM 940 and triethanolamine were obtained from Macklin Biochemical Technology Co., Ltd. (Shanghai, China). DPPH and vitamin C were sourced from Aladdin Biochemical Technology Co., Ltd. (Shanghai, China). Recombinant human epidermal growth factor (yeast) gel (EGF) was purchased from Huanuowei Gene Pharmaceutical Co., Ltd. (Guilin, China). *Staphylococcus aureus* (*S. aureus*, ATCC25923) and *Escherichia coli* (*E. coli*, ATCC25922) were obtained from American Type Culture Collection. Dulbecco’s modified Eagle’s medium (DMEM), fetal bovine serum, penicillin and streptomycin were purchased from Gibco (Waltham, MA, USA). A Cell Counting Kit-8 (CCK-8) was purchased from Biosharp. Flow cytometry kits were purchased from Becton, Dickinson and Company (Waltham, MA, USA), while an ROS Assay Kit and streptozotocin (STZ) were obtained from Beyotime Biotechnology Co., Ltd. (Shanghai, China). A Calcein-AM/PI Double Stain Kit was purchased from Yeasen Biotechnology Co., Ltd. (Shanghai, China).

### 3.2. Synthesis of BSP/BER Hydrogel

To prepare the BSP/BER hydrogel, CBM 940 was first dissolved in deionized water. After defoaming, BSP and BER were added to the CBM 940 solution, and electromagnetic stirring was employed for 12 h to ensure complete dissolution. The pH of the solution was adjusted to 7 using triethanolamine, and continuous stirring was maintained until a clear and transparent hydrogel was obtained [27]. A series of hydrogels were prepared by adding different amounts of BSP and BER, as shown in Table 1, and they were named HBSP/BER1, HBSP/BER2, LBSP/BER1 and LBSP/BER2.

### 3.3. Characterization of BSP/BER Hydrogel

#### 3.3.1. Scanning Electron Microscopy (SEM)

The microstructure of the hydrogel was observed via SEM. In brief, a portion of the freeze-dried hydrogel samples was placed on the sample table and sputtered with gold (30 mA, 20 s) and then observed with a Hitachi SU8010 (Tokyo, Japan) operating at 10 kV and 10 μA.

#### 3.3.2. Fourier Transform Infrared Spectroscopy (FTIR)

After thoroughly grinding the sample with KBr, a uniform compression pellet was prepared and the FTIR spectra were collected using a Thermo Scientific Nicolet iS50 FTIR spectrometer (Waltham, MA, USA) within the range of 500–4000 cm^−1^ at a scanning resolution of 2 cm^−1^.

#### 3.3.3. Viscosity and Rheology Characteristics of Hydrogel

An NDJ-5S rotary viscometer was employed for the viscosity measurement. After transferring the sample to the measuring cup, the viscosity at a room temperature of 25 °C was measured using rotor six, with three measurements for each sample.

The rheology characteristics of the hydrogel were measured using an Anton Paar MCR 302 Rotational Rheometer (Austria). At a temperature of 25 ± 1 °C, the storage modulus G′ and loss modulus G″ of the hydrogel were measured in the frequency range of 0.1–10 Hz (0.01% strain) using a parallel plate with a diameter of 50 mm.

#### 3.3.4. Water Loss Rate

The hydrogel samples were accurately weighed and placed in centrifuge tubes. These tubes were then placed in a temperature-controlled oven at 37 °C, and the loss of moisture was monitored over time. The cumulative weight loss rate was determined using the following formula:

(1) $$Water\;loss\;rate\left(\%\right)=\frac{W_{0}-W_{t}}{W_{0}}\times 100\%$$

where W_0_ and W_t_ were the initial mass of the hydrogel and its mass after time t, respectively.

#### 3.3.5. BER Release Behavior from Hydrogel

A conical tube containing 20 mL of PBS buffer solution and 4 g of hydrogel sample was placed in a shaker at 37 °C and a speed of 100 r/min. At specific time intervals (1, 3, 6, 12, 24, 36, and 48 h), 1 mL of supernatant was collected from the tube, and the tube was replenished with 1 mL of PBS buffer. The absorbance of the sample at 340 nm was measured using an Epoch 2 microplate reader (UAS). The concentration of BER was determined by calculating it based on the concentration standard curve of a BER solution prepared in PBS buffer. Subsequently, a cumulative release curve was plotted.

### 3.4. In Vitro Assessments

#### 3.4.1. Cell Culture

Mouse RAW 264.7 macrophages (RAW 264.7, Chinese Academy of Sciences Cell Bank, Shanghai, China), mouse fibroblasts (L929, Chinese Academy of Sciences Cell Bank, Shanghai, China), and human umbilical vein endothelial cells (HUVEC, Chinese Academy of Sciences Cell Bank, Shanghai, China) were grown in a modified culture medium composed of 90% DMEM, 10% fetal bovine serum, and 1% penicillin–streptomycin. The culture conditions were maintained at 37 °C with a 5% CO_2_ atmosphere.

#### 3.4.2. Cytocompatibility Experiments

The cytotoxicity evaluation of each hydrogel was carried out using L929 cells and HUVEC cells in a CCK-8 assay. Culture medium containing L929 or HUVEC cells was seeded on 96-well plates at 1 × 10^4^/well. Meanwhile, the extract solution (4 mg/mL) was prepared via immersion of the hydrogel in DMEM for 24 h at 37 °C, and then 100 μL of hydrogel extract was added to each well before incubation for 24 and 48 h. The culture medium without cells added was used as a blank, and the cells without treatment using hydrogel extracts as the control. Finally, CCK-8 was added to the plates, and the optical density of the culture plate solution was measured at 450 nm using a microplate reader. The calculation of the cell viability was as follows:

(2) $$Cell\;viability\left(\%\right)=\frac{\left(O_{test}-O_{blank}\right)}{O_{control}}$$

where O_test_, O_blank_ and O_control_ were the absorbance of the test, blank, and control, respectively.

For the live/dead assay, L929 or HUVEC cells were evenly spread in 12-well plates at a density of 5 × 10^4^ cells/well and placed in an incubator with 5% CO_2_ at 37 °C until the cells adhered completely. The cells in each well were stained according to the instructions for the Calcein-AM/PI Double Stain Kit. The results of the fluorescence staining were obtained under a Nikon DS-Ri2 inverted fluorescence microscope (Tokyo, Japan).

#### 3.4.3. Hemolysis Assessment

The in vitro blood compatibility of the BSP/BER hydrogel was assessed by directly placing 2% red blood cell suspension in contact with the hydrogel samples at 37 °C. The absorbance of the supernatant at 545 nm was measured spectrophotometrically. For comparison, blood samples were mixed with deionized water and physiological saline as the positive and negative controls, respectively. The calculation of hemolysis rate was as follows:

(3) $$Hemolysis\;ratio\left(\%\right)=\frac{{OD}_{s}-{OD}_{n}}{{OD}_{p}-{OD}_{n}}\times 100\%$$

where OD_n_, OD_p_, and OD_s_ were the optical density values of the negative control, positive control, and sample, respectively.

#### 3.4.4. Determination of Cellular Glucose Consumption

The glucose level in the supernatant of L929 cells was measured to assess the glucose consumption ability of the cells. L929 cells were cultured in a 96-well plate at a density of 0.8 × 10^4^ cells/well and incubated for 48 h with different hydrogel extracts after the cells adhered. Subsequently, the glucose content in each well was detected using a glucose detection kit, and the cell viability was detected using a CCK-8 assay. The culture medium without cells added was used as a blank, and the cells without treatment using hydrogel extracts served as the control. The calculation of glucose consumption was based on the following equation:

(4) $$Glucose\;consumption\left(mmol/L\right)=\frac{{Glu}_{blank}-{Glu}_{test}}{Cell\;viability}\times 100\%$$

where Glu_blank_ represents the absorbance of the content of glucose in the blank group and Glu_test_ represents the content of glucose in the hydrogel experimental group.

#### 3.4.5. Cell Migration Assay

L929 or HUVEC cells were seeded evenly in 12-well plates at a density of 1 × 10^5^ cells/well and incubated at 37 °C with 5% CO_2_ for 24 h until the cells adhered completely. A scratch was created in the plate using a pipette tip, and this was followed by two washes with PBS. Hydrogel extract was added to the plate, and the scratch was observed and photographed at 0 h and 24 h.

#### 3.4.6. Antioxidant Properties Assay

The extracellular antioxidant activity of the hydrogel was evaluated using a DPPH scavenging test. Different BSP/BER hydrogel extracts (4 mg/mL) were mixed with a DPPH ethanol solution, with vitamin C as the positive control and deionized water as the negative control. After incubating for 30 min in the dark, the absorbance at 516 nm in the supernatant was measured using a microplate spectrophotometer. The percentage of DPPH free radical clearance was calculated using the following equation:

(5) $$DPPH\;inhibition\left(\%\right)=1-\frac{A_{2}-A_{1}}{A_{0}}\times 100\%$$

where A_0_ was the absorbance of ethanol with DPPH, A_1_ was the absorbance of the sample mixed with ethanol, and A_2_ was the absorbance of the sample mixed with DPPH.

In the investigation of the intracellular antioxidant properties of the hydrogel, L929 cells were cultured in 12-well plates at a density of 5 × 10^5^ cells/mL until they adhered. The cells were then pre-stimulated with hydrogel extracts for 12 h. Afterwards, 900 μM H_2_O_2_ was added to treat the cells for an additional 4 h. The untreated cells served as the control group, while the cells treated only with H_2_O_2_ served as the model group (MOD). The cells were stained with DCFA–DA, and the fluorescence results were observed using an inverted fluorescence microscope. The staining results were quantitatively analyzed using a Becton Dickinson FAC Scan flow cytometer (Franklin Lakes, NJ, USA).

#### 3.4.7. Anti-Inflammatory Activity Assay

The RAW264.7 cells at exponential growth were seeded in 6-well plates at a density of 5 × 10^5^ cells/well. The cells were pretreated with culture medium containing hydrogel extracts for 2 h. Afterwards, they were stimulated with lipopolysaccharide (LPS) at a concentration of 1 μg/mL for 24 h. The untreated cells served as the control group, while the cells treated only with LPS served as the model group (MOD). The cell supernatant was collected, and the concentrations of tumor necrosis factor (TNF)-α, monocyte chemotactic protein (MCP)-1, and interleukin (IL)-6 were measured using flow cytometry.

#### 3.4.8. Antibacterial Activity Assay

The antibacterial effects of the hydrogel were assessed using liquid medium turbidity assays [48]. A total of 50 mg of hydrogel was added to 5 mL of Luria-Bertani (LB) liquid medium, which contained bacteria (*S. aureus*, or *E. coli*) at a concentration of 1 × 10^6^ CFU/mL in the logarithmic growth phase. Untreated bacteria were used as the control group. The mixture was then incubated in a 37 °C thermostat, and the absorbance of the bacterial suspensions at 600 nm was measured using an Epoch 2 microplate reader. Furthermore, 100 μL of the bacterial suspension was evenly coated on a medium plate, and the colonies were observed after incubation at 37 °C for 24 h. The antibacterial activity of the hydrogel was calculated using the following formula:

(6) $$Antibacterial\;activity=\frac{{OD}_{con}-{OD}_{test}}{{OD}_{con}}\times 100\%$$

where OD_con_ represents the absorbance of the bacterial liquid in the control group and OD_test_ represents the absorbance of the dressing experimental group.

### 3.5. In Vivo Assessments

#### 3.5.1. Diabetic Chronic Wound Healing Assay

ICR mice (male, 6–8 weeks old, weighing 20–30 g) were obtained from the Animal Experiments Center of Zhejiang Chinese Medical University. Diabetes mellitus was induced in the ICR mice by injecting them with STZ at a dosage of 120 mg/day kg body weight for 2 consecutive days, and the blood glucose levels of mice were measured on the 3rd day after the injection was completed. The mice were considered diabetic if the nonfasted glycemia was higher than 16.7 mmol/L.

Then, the mice were randomly divided into 5 groups of control (negative control), EGF (positive control), HBSP/BER1, HBSP/BER2, LBSP/BER2 and LBSP/BER2 group (n = 6). After shaving the hair, a full-thickness skin wound with a diameter of 10 mm was made on the dorsal side of the diabetic mice under anesthesia using atropine and Zoletil 50. The wounds in the different groups of mice were treated with the corresponding hydrogel, and the dressings were changed every two days. Photos of the wounds were taken on the 4th, 7th, 10th, and 14th day after the injury, and the wound closure was calculated using the following equation:

(7) $$Wound\;Closure\left(\%\right)=\frac{A_{i}-A_{d}}{A_{i}}\;$$

where A_0_ denoted the initial wound area and A_t_ represented the wound area at a specific time interval.

#### 3.5.2. Histopathological Evaluation

On the 14th day post-injury, the mice were euthanized, and the tissue samples were collected and fixed in a 4% paraformaldehyde solution. The specimens were subsequently embedded in paraffin and sliced using a microtome. The sections were stained with hematoxylin and eosin (H&E) as well as Masson’s trichrome stain. The sections were examined under a Nikon light microscope (Tokyo, Japan) and captured using a camera.

### 3.6. Statistical Assay

All the results were presented as the mean ± standard deviation (SD). SPSS Statistics software version 20 was used for the statistical analysis of the data. A one-way ANOVA test was used to determine possible significant differences between the dependent and independent samples. A *p*-value < 0.05 was considered to be statistically significant (* for *p* < 0.05 and ** for *p* < 0.01).

## 4. Conclusions

In conclusion, this study developed a BSP/BER hydrogel that demonstrated promising effects in promoting the healing of DWs. The hydrogel exhibits antibacterial, anti-inflammatory, and antioxidant properties, primarily through its ability to effectively inhibit the growth of *E. coli* and *S. aureus* as well as to reduce the production of pro-inflammatory factors and ROS. Furthermore, the hydrogel also possesses the potential for local hypoglycemia and can alleviate the wound deterioration caused by local hyperglycemia to a certain extent. In animal experiments, DWs treated with the BSP/BER hydrogel exhibited a significantly accelerated healing rate and superior healing quality, with LBSP/BER2 standing out as the most effective formulation. In summary, the BSP/BER hydrogel held tremendous potential for the treatment of DWs. Its unique properties, along with the encouraging experimental results, make it a promising candidate for further investigation and potential clinical applications.

## Figures and Tables

**Table 1:** The sample codes and compositions of the BSP/ BER hydrogels.

Sample Code	BSP (g)	BER (g)	Carbomer 940 (CMB 940) (g)	Water (mL)
HBSP/BER1	1.00	0.05	0.25	50
HBSP/BER2	1.00	0.25	0.25	50
LBSP/BER1	0.50	0.25	0.25	50
LBSP/BER2	0.50	0.125	0.25	50

**Table 2:** Results of the FTIR analysis of the raw materials in the BSP/BER hydrogels.

Materials	Absorption Peak	Chemical Group
BSP	3420 cm^−1^	-OH
	2819 cm^−1^	-CH
	1732 cm^−1^	-COOH
	1126 cm^−1^	-C-O-C-
	872 cm^−1^	β-glucose
	764 cm^−1^	Mannose residues
BER	3420 cm^−1^	-OH
	2845 cm^−1^	-CH
	1597 cm^−1^, 1507 cm^−1^	Benzene ring skeleton
CBM940	3446 cm^−1^	-OH
	2959 cm^−1^	-CH
	1716 cm^−1^	-COOH

## Data Availability

Data are contained within the article.
